# A pilot study of digital recording of edentulous jaw relations using a handheld scanner and specially designed headgear

**DOI:** 10.1038/s41598-018-27277-5

**Published:** 2018-06-12

**Authors:** Weiwei Li, Qiufei Xie, Yong Wang, Yuchun Sun

**Affiliations:** 1 Center of Digital Dentistry, Peking University School and Hospital of Stomatology, Beijing, China; 2 Department of Prosthodontics, Peking University School and Hospital of Stomatology, Beijing, China; 3 National Engineering Laboratory for Digital and Material Technology of Stomatology, Beijing, China; 4 Research Center of Engineering and Technology for Digital Dentistry of Ministry of Health, Beijing, China; 5 Beijing Key Laboratory of Digital Stomatology, Beijing, China; 6 Center for Oral Functional Diagnosis, Treatment and Research, Peking University School and Hospital of Stomatology, Beijing, China

## Abstract

The present study aimed to establish and evaluate a method for recording edentulous jaw relations digitally without occlusal bases, using a handheld scanner and specially designed headgear. The headgear maintained the mandibular position. Ten edentulous patients’ upper (U) and lower edentulous jaw models (L) were prepared and scanned. A handheld scanner was used to capture the labial alveolar ridge mucosa relations in the upper and lower anterior arches directly (Dr). U and L were registered to Dr (test group). Complete dentures of patients in the intercuspal position were used to construct the relationship between U and L (control group). Differences in jaw relations in the test and control groups, in terms of vertical difference, displacement and rotation of the anterior and posterior, and displacement and rotation of the left and right were assessed using the Hotelling’s T^2^ test. The differences in the mean values and the mean of the absolute values of the jaw relations between groups were not statistically significant (*P* = 0.331) and significant (*P* = 0.016), respectively. Our findings show that it is possible to make digital recording of edentulous jaw relations by using a handheld scanner and headgear.

## Introduction

Jaw relations of edentulous individuals should be established and recorded for making complete dentures^1–4^. Various studies have considered differing techniques for recording jaw relations^5–12^.

The intraoral relation between the upper and lower edentulous alveolar ridges are typically recorded using occlusal bases^2–4^. The upper and lower bases are respectively seated stably on upper and lower edentulous models. Then, the intraoral jaw relations are transformed into extraoral models fixed by positioning and locking structures, which are generated by means of solidified bite registration material on the occlusal rim. The occlusal rims, including fullness and occlusal planes of the upper rim, and the height of the lower rim are adjusted to obtain a record of the vertical dimension by adding or subtracting wax. Then, the horizontal relationship is recorded by the solidified bite registration. Both require proficient manipulation of the bite registration material and technical guidance of the mandibular position to ensure that the condyle is in a suitable position for clinical recording of the jaw relations in edentulous patients.

This traditional method of recording jaw relations is challenge for young dentists^13^, as complete mastery requires experience. Simplified methods have been suggested^14–17^. Although traditional techniques for production of complete dentures have long been used^18^, digital techniques have come to be applied to record jaw relations, allowing greater efficiency, accuracy, and automation^19–22^. Use of a digital Gothic arch tracer^23,24^ or mandibular movement trajectory tracer^25,26^ allows clear verification of the mandibular position clearly on a computer monitor. Nevertheless, without three-dimensional (3D) data of the upper and lower models in the tracing system, the 3D construction of jaw relations is not possible. The application of the SICAT function^21^ has enabled dentists to observe the jaw movement dynamically; however, one cone-beam computed tomography scan is needed.

Thus, digital methods for recording jaw relations in edentulous jaws, without radiation, are needed. Traditionally, occlusal bases are used to record the relationship between the upper and lower jaw, aided by an intraoral stop position in the mandible. When producing CAD/CAM complete dentures, occlusal bases are scanned externally^27–29^, in a manner similar to the conventional process of recording the jaw relations of edentulous individuals. Many studies^30–32^ have shown that 3D scanning of occlusal bases is effective. However, we sought to develop a new approach for recording jaw relations digitally, without the need for occlusal bases, and here present a pilot study of the new method, which uses an extraoral stop position in the mandible to record the relations in edentulous jaws, using headgear and a handheld 3D scanner.

## Materials and Methods

### Patient enrolment and model preparation

The study was approved by the Bioethics Committee of the Stomatological Hospital of Peking University, Beijing, China (PKUSSIRB-2013010) and was carried out in accordance with approved guidelines for human subjects research. The procedures and risks involved with participation in this study were discussed with the volunteers, and written informed consent was obtained from all participants, and we got written permission from one participant to use the identifying photographs in our online open-access publication. The participants were selected from the outpatient clinic of Department of Prosthodontics at Peking University School and Hospital of Stomatology according to the following inclusion and exclusion criteria:

Patients were male or female, completely edentulous patients, aged 65–80 years, who were satisfied with their complete dentures. Patients had to be able to understand and cooperate with the requirements of the study. They had Atwood’s degree II or III absorption of the alveolar ridge and Angel’s class I jaw relations, and had good neuromuscular control. Patients were excluded if they had temporomandibular joint disorders, mental illness, highly resorbed ridges or systemic disorders.

A pilot study was performed on ten patients (four male and six female patients, aged 71.6 ± 8.0 years) selected based on the above inclusion and exclusion criteria. Based on the requirements of a prosthodontics textbook^4^, maxillary and mandibular impressions of the patients were made following the two-step impression method, and a gypsum model was then poured into a model base.

### Design, manufacture, and usage of headgear prototype

**Figure 1:**
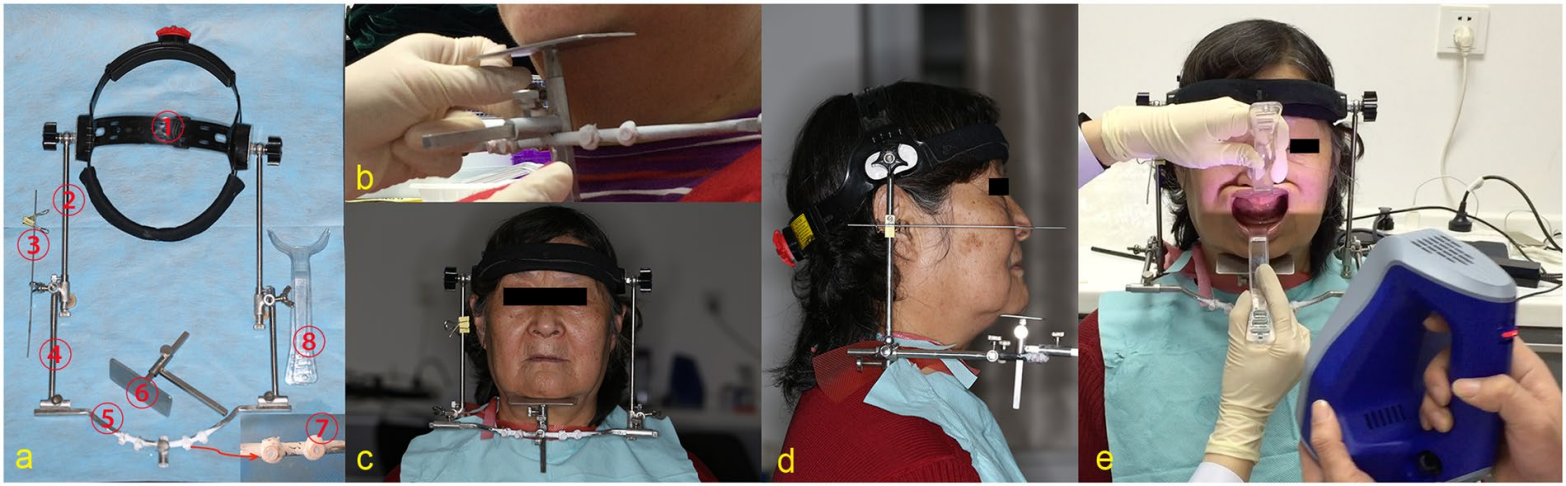
The headgear and data acquisition of test group. (**a**) The structure of the self-developed headgear and the retractor. (**b**) Adjustment of the chin plate. (**c**) Front view of the patient. (**d**) Lateral view of the patient. (**e**) 3D maxillomandibular relation record.

We designed and fabricated headgear that could maintain an extraoral stop position for directly recording the digital jaw relations. The headgear consisted of seven parts (Fig. 1a):

1. Head cap: This was fitted onto the head of patients and linked to the lower structure of the headgear. Its size could be adjusted for the individual head size.
2. Supero-inferior slider: The connection between the head cap and the supero-inferior slider allowed the slider to rotate forwards to backwards.
3. Ala-tragus line guide-tip: This points to the ala-tragus line of the patient located perpendicular to the supero-inferior slider.
4. Antero-posterior slider: This is perpendicular to the supero-inferior slider and its location could be adjusted from above to below to adapt to the length of the patient’s face.
5. Left-to-right slider: This connects the front tip of the antero-posterior slider. Its width could be adjusted to make the bilateral supero-inferior sliders parallel.
6. Chin plate: This is located in the center of the left-to-right slider and parallel to the alar-tragus line guide-tip; the flat plane of the chin was connected vertically to the left-to-right slider and could be adjusted upward and downward, to the front and rear. It touched the soft tissue and was used to maintain the extraoral stop position in the mandible (Fig. 1b).
7. Positioning cylinder: Positioning cylinders of the same size (base plane diameter of 6 mm, height of 5 mm) were designed and prepared using a 3D resin printer (EnvisionTEC, Gladbeck, Germany). Four positioning cylinders were adhered to the left-to-right slider. The positioning cylinders were used as common regions for registering the maxillomandibular 3D data to the facial 3D data.

### 3D data acquirement aided by the headgear

The dental chair was adjusted to allow the patient to sit erect and look straight ahead, with the headgear fitted onto the head, with the ala-tragus line guide-tip pointing to the patient’s ala-tragus line, and vertical to the supero-inferior slider (Fig. 1c,d). The height and width of the headgear were adjusted to the patient’s frame by adjusting the antero-posterior slider and the left-to-right slider. Finally, depending on the vertical dimension decided by applying the mandibular postural position and referencing to the vertical dimension of the complete denture, the chin plate was adjusted and fixed in the anterior, posterior, left, and right directions.

**Figure 2:**
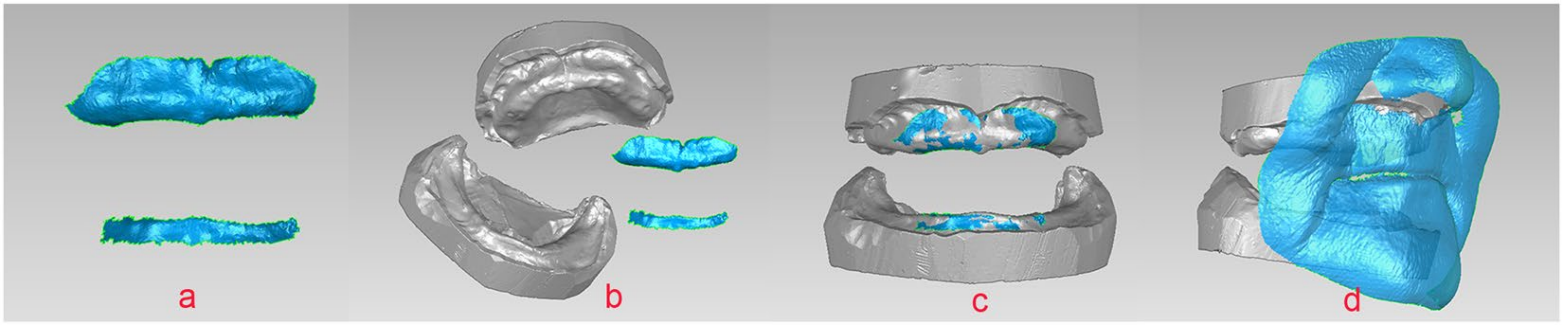
The 3D construction of the jaw relations in the test group. (**a**) Jaw relation record of the upper and lower edentulous jaws (Dr). (**b**) U, L, and Dr data were imported into Geomagic 2012. (**c**) 3D construction of direct digital jaw relation record. (**d**) 3D data of the lower facial one-third (Fr) and models of jaw relation.

The horizontal relations were determined by instructing the patients to swallow and place their chin on the chin plate, or to hold the tip of their tongue back toward the soft palate while maintaining contact with the chin plate^33^. After the jaw relations were determined, the chin plate was placed in appropriate contact with the soft tissue of the chin. The midline, lines of the angulus oris, and inferior margin of the upper lip were referenced to the lower one-third of the 3D facial data (Fr) and recorded using the Artec Spider handheld scanner (Artec, Orlando, FL, USA). Using the retractor, the upper lip was pulled gently forward and upward, and the lower lip forward and downward (Fig. 1e). The relations between the upper and lower edentulous jaw (Dr) was directly recorded using the handheld scanner (Fig. 2a). The 3D data were stored in STL format.

### 3D construction of jaw relation

The Activity880 (SmartOptics, Bochum, Germany) was used to perform a 3D scan of the upper and lower edentulous models. The upper and lower 3D data were stored in STL format.

The U, L, and Dr were imported into Geomagic 2012 software (Raindrop Geomagic, Morrisville, NC, USA). Based on the common region of the alveolar crest and secondary stress-bearing area in the anterior arch, and applying “N points alignment” and “best fit” in Geomagic 2012, U and L were registered to Dr (Fig. 2b,c). Fr and Dr data could be registered based on the positioning cylinder in the headgear (Fig. 2d). The midline, lines of the angulus oris, and inferior margin of the upper lip were deduced from the Fr data. Thus, the jaw relations of the test group were established.

**Figure 3:**
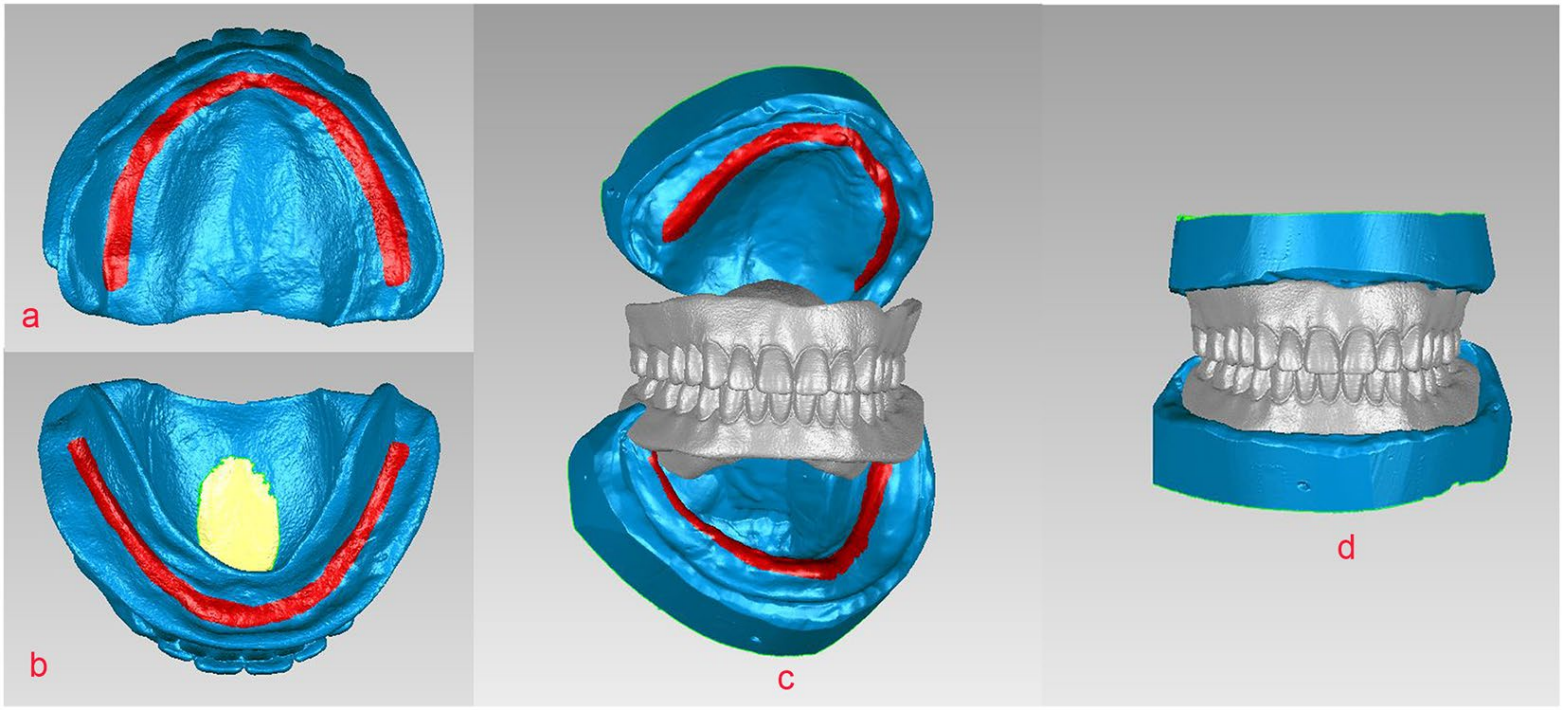
The 3D construction of the jaw relations in the control group. (**a**) Tissue surface of the upper denture with the red common region used for registration. (**b**) Tissue surface of the lower denture with the red common region used for registration. (**c**) Models and complete dentures were imported into Geomagic 2012. (**d**) 3D construction of models of the jaw relations of the complete denture.

The vertical and horizontal dimensions of the complete denture were determined by a dentist with >10 years of experience in making complete dentures. Patients were satisfied with their complete dentures. After confirming the accuracy of these dentures with the dentist and patients, these dentures were used as controls. The upper and lower complete dentures were then fixed and comprehensively scanned extraorally in the intercuspal position using the handheld 3D scanner (Cr data). Based on the common region of the primary and secondary stress-bearing area of the upper and lower dentures and models, U, L, and Cr data were imported and constructed in the Geomagic 2012 to obtain the models with the jaw relations of the complete dentures (control group) (Fig. 3).

### Measurement

#### Establishment of the coordinate system

**Figure 4:**
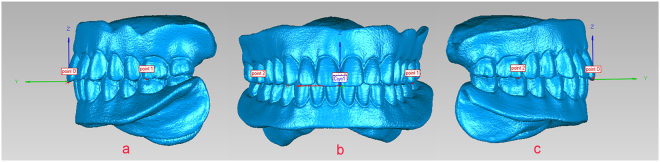
Establishment of the coordinate system (**a**) Left view. (**b**) Front view. (**c**) Right view.

The registration data of the control group were imported into Geomagic 2012. The lines of the maxillary central incisor edge and the midline were selected in the upper complete denture, and the point of intersection, O, was determined. The left and right second premolar buccal cusps of the upper complete denture were defined as points 1 and 2. The XOY plane was determined based on points 1, 2, and O, with point O being the coordinate origin. The X-axis was parallel to the line between points 1 and 2. Observation of the coordinate system was established based on Descartes’ rule of signs (Csys1): the X-axis of the coordinate system was horizontal left/right, the Y-axis was horizontal anterior/posterior, and the Z-axis was vertical (Fig. 4).

For the lower coordinate system, in the registration data of the control group, Csys1 was copied and superimposed on the lower model of the edentulous jaw. This local coordinate system was termed Csys2, and Csys2 was registered to the lower model of the test group, termed Csys3. Csys2 and Csys3 in the lower model of the control and test groups, respectively, represented the differences in vertical dimensions and horizontal relations.

#### Measurement of jaw relations

The jaw relations of the test group were registered in the control group based on the common U region in Geomagic 2012. The lower coordinate system features in the lower jaw models were extracted and imported to Imageware13.2 reverse engineering software (Siemens, Munich, Germany). The differences in the jaw relations between the test and control groups were analyzed based on differences in Csys3 and Csys2.

Differences in the vertical dimension (VD) were displacements in point O on the Z-axis between Csys3 and Csys2 in Csys1 (Csys2 was the same as Csys1). The differences in the horizontal dimension consisted of horizontal displacement to the anterior and posterior (DAP), horizontal rotation to the anterior and posterior (RAP), horizontal displacement to the left and right (DLR), and horizontal rotation to the left and right (RLR).

Antero-posterior horizontal displacement was the difference in the position of point O on the Y-axis between Csys3 and Csys2 in Csys1. Horizontal rotation to anterior and posterior was the angle between the projection of the Y-axis of Csys3 in the YOZ plane of Csys1 and the Y-axis of Csys2. Horizontal displacement to the left and right was the difference in the position of point O on the X-axis between Csys3 and Csys2 in Csys1. Horizontal rotation to the left and right was determined from the angle between the projection of the X-axis of Csys3 in the XOZ plane of Csys1 and the X-axis of Csys2.

The vertical difference and horizontal displacements were obtained using the software’s “measure, distance, and between points” command, and horizontal rotation was determined by applying the “measure, angle/tangent direction, and between curve tangents” command. The rotation value was positive if the direction of rotation was in the positive direction on the Z-axis, otherwise the rotation value was negative.

The direct digital recording of the jaw relations was performed three times per patient, with an interval of 5 min. The vertical and horizontal differences in the 3D construction of the jaw relations were analyzed in the test group as compared with the jaw relations of complete artificial dentures fixed in the intercuspal position.

### Statistical analysis

All statistical analyses were performed with SPSS 20 statistical software (SPSS Inc., Cary, NC, USA). A normal distribution of our data was shown using the normality test. The differences in the jaw relations (in terms of VD, DAP, RAP, DLR, and RLR), in three directions, X, Y, and Z, between the test and control groups, were analyzed using Hotelling’s T^2^ test. *P* values < 0.05 were considered statistically significant.

## Results

Table 1 shows the results of the 3D analysis of the models, indicating the jaw relations in ten patients. The 95% confidence intervals (CIs) of the VD, DAP, RAP, DLR, and RLR were −1.791 to 0.520, −0.275 to 1.053, −2.38 to 0.99, −0.588 to 0.946, and −1.04 to 0.50, respectively.

**Table 1:** Differences in jaw relations between the test and control groups.

No.	Times	VD (mm)	Horizontal			
			AP		LR	
			DAP (mm)	RAP (°)	DLR (mm)	RLR (°)
1	1	−5.486	−1.239	−3.04	2.493	−0.09
	2	0.595	1.678	−2.56	1.379	−0.58
	3	−1.655	0.073	−4.76	1.605	0.71
2	1	−0.744	0.190	−5.43	−0.906	−0.51
	2	2.570	4.363	3.37	−4.898	−2.15
	3	2.130	0.144	1.83	0.277	−0.16
3	1	1.049	−0.481	−4.90	1.301	3.62
	2	2.924	3.058	5.55	0.072	4.16
	3	−1.107	1.934	−10.21	−0.088	1.80
4	1	−2.052	2.233	−1.01	−1.530	−0.10
	2	1.126	1.177	2.08	−3.225	−1.33
	3	0.541	0.637	4.36	−1.358	−0.44
5	1	1.482	2.475	8.31	2.677	0.17
	2	2.672	1.488	3.64	−0.091	1.13
	3	1.175	1.390	5.52	0.661	0.77
6	1	−4.504	−1.375	−3.33	−0.350	0.66
	2	−5.823	−3.922	−4.31	−2.586	−0.03
	3	−5.476	−2.698	−7.15	0.197	1.02
7	1	4.452	0.046	3.06	1.200	−2.36
	2	2.391	1.132	−2.54	2.361	−4.33
	3	5.133	1.523	0.97	2.052	−0.60
8	1	−2.665	−0.161	−2.07	1.214	−3.00
	2	−0.130	0.187	1.33	−0.891	−0.01
	3	−3.191	−0.153	−2.72	−0.234	0.88
9	1	2.061	0.208	5.67	−1.365	−5.65
	2	−1.868	−1.421	0.45	−1.899	−0.99
	3	−2.435	−2.833	1.70	−1.419	−2.35
10	1	−4.400	−0.035	−2.96	0.514	0.01
	2	−3.511	2.034	−5.94	5.091	−0.84
	3	−4.318	0.025	−5.79	3.130	2.56
mean		−0.636	0.389	−0.70	0.179	−0.27
95% CIs		−1.791:0.520	−0.275:1.053	−2.38:0.99	−0.588:0.946	−1.04:0.50

VD: vertical dimension; AP: anterior posterior; LR: left and right; DAP: displacement in anterior and posterior; RAP: rotation in anterior and posterior; DLR: displacement to left and right; RLR: rotation to left and right.

Table 2 shows the mean values and the mean absolute values of the three measurements per patient. The differences in the mean vertical and horizontal values of the groups were not statistically significant (Hotelling’s T2 = 8.563; *P* = 0.331). However, the differences of the mean vertical and horizontal absolute values of the groups were statistically significant (Hotelling’s T2 = 45.697; *P* = 0.016). Mean absolute values will avoid the offset effects of positive and negative data, which represent the positive and negative direction of the axes, making it easier to detect significant differences.

**Table 2:** Comparison of the mean values (and the mean absolute values) in vertical dimensions and horizontal relationships in patients, by Hotelling’s T^2^ test.

Patient No.	VD (mm)	Horizontal				Hotelling’s T^2^	*P* value
		AP		LR			
		DAP (mm)	RAP (°)	DAP (mm)	RAP (°)		
1	−2.182 (2.579)	0.171 (0.997)	−3.45 (3.45)	1.825 (1.825)	0.01 (0.46)	8.563 (45.697)	0.331 (0.016)
2	1.318 (1.814)	1.566 (1.566)	−0.08 (3.54)	−1.842 (2.027)	−0.94 (0.94)		
3	0.956 (1.693)	1.504 (1.824)	−3.19 (6.89)	0.428 (0.487)	3.19 (3.19)		
4	−0.128 (1.240)	1.349 (1.349)	1.81 (2.48)	−2.038 (2.038)	−0.62 (0.62)		
5	1.776 (1.776)	1.784 (1.748)	5.83 (5.83)	1.082 (1.143)	0.69 (0.69)		
6	−5.268 (5.268)	−2.665 (2.665)	−4.93 (4.93)	−0.913 (1.044)	0.55 (0.57)		
7	3.992 (3.992)	0.901 (0.901)	0.50 (2.19)	1.871 (1.871)	−2.43 (2.43)		
8	−1.995 (1.995)	−0.042 (0.167)	−1.15 (2.04)	0.029 (0.780)	−0.71 (1.30)		
9	−0.748 (2.122)	−1.349 (1.487)	2.61 (2.61)	−1.561 (1.561)	−3.00 (3.00)		
10	−4.076 (4.076)	0.675 (0.698)	−4.90 (4.90)	2.912 (2.912)	0.58 (1.14)		

VD: vertical dimension; AP: anterior posterior; LR: left and right; DAP: displacement in anterior and posterior; RAP: rotation in anterior and posterior; DLR: displacement to left and right; RLR: rotation to left and right.

## Discussion

Using the handheld scanner and specially designed headgear, we have accomplished digital recording of the jaw relations of edentulous patients, directly, without the use of occlusal bases. Thus, for fabrication of the CAD/CAM complete dentures, the production process is simplified and the number of appointments can be reduced.

Traditionally, the jaw relations in edentulous individuals are recorded in the clinic using occlusal bases, and an upper model, upper occlusal base, lower model, lower occlusal base, and the labial/buccal surface of the models fixed by occlusal bases must be scanned individually. Thereafter, the ICP algorithm of the common labial/buccal surface that is used for 3D construction of the edentulous jaw model with the jaw relations is implemented^31^. 3D construction of edentulous jaw relations by means of commercial complete denture systems, such as Avadent, Dentaca, and 3Shape, also use occlusal bases that are scanned extraorally^27–29^. We digitally recorded the edentulous jaw relations aided by extraoral headgear, which maintained the jaws relations and a handheld 3D scanner, by which we could obtain a record of the anterior arch of the upper and lower jaws.

The ideal 3D scanner needs to obtain the morphology of the membrane located in the anterior edentulous ridge of upper and lower jaw, and the lower one-third of facial data simultaneously^34^. A facial 3D scanner, oral 3D scanner, and handheld 3D scanner could potentially be used in such a study. A facial 3D scanner, such as 3D Face Scanner and the 3dMD head system, can obtain a facial 3D point-cloud and texture information of patients, including the eyes, nose, mouth, and part of the ears, in 1.5–800 milliseconds with an accuracy of 100–200 μm^35,36^; for the membrane in the anterior edentulous ridge of the upper and lower jaws with depth of field, the 3D data obtained with a facial 3D scanner are inappropriate. A dental oral scanner, such as the CEREC AC Bluecam and 3Shape TRIOS, has a higher accuracy of 20–30 μm, and a smaller single field of view. In contrast to full dentition, the characteristic geometric morphological area, with obvious curvature changes, are lacking in the edentulous alveolar ridge surface, which inevitably affects the accuracy of the automated stitching of 3D multi-viewpoint images^37^. The accuracy of a handhold 3D scanner, including the Artec Spider and the Creaform portable scanner, is 50–100 μm, which is between that of the oral scanner and facial scanner. The 3D data of the membrane in the anterior edentulous ridge of the upper and lower jaws were used for 3D construction of the jaw relations and that of the lower one-third facial were used to extract the features of the midline, the lines of the angulus oris, and the inferior margin of the upper lip. Therefore, higher accuracy, larger scanning range, and shorter scanning time are necessary, and thus, the handheld 3D scanner was considered the better choice for our study.

To record digital jaw relations, we used a handheld 3D scanner to obtain data of the upper and lower edentulous anterior ridge mucosa, with the help of an retractor, which gently pulled the upper lip forward and upward, and the lower lip forward and downward. The curvature of the anterior ridge is relatively large, and it can be considered for registration of the whole dental arch. Based on the common region of the alveolar crest and secondary stress-bearing area in the anterior arch, the upper and lower models were registered to the digital jaw relation record to construct jaw relations. The horizontal anterior and posterior rotation was relatively large in the 3D construction of the jaw relations. This may be because the lower model rotates downward and backward; therefore, differences in the vertical dimension tend to increase. The more 3D data available for the alveolar crest and the secondary stress-bearing area in the anterior arch, the more the accuracy of the 3D construction of the jaw relations for the common region increased and the rotation values decreased.

The retractors were obtained from the lip-press plate used in dental photography. In order to minimize the influence of the retractor used by the dentist when determining jaw relations, the upper and lower lips were retracted gently. The mandible might be pulled somewhat downward, increasing the vertical dimension, while movement of the head might influence the accuracy of the 3D scanning during this process. Therefore, care should be taken to verify jaw relations when exposing the upper and lower edentulous alveolar ridges mucosa.

We here demonstrated the direct digital recording of edentulous jaw relations aided by headgear and a handheld 3D scanner. The chin plate of the headgear was gently contacted to the soft tissue of the chin, in order to reduce the influence of the process of adjusting and fixing the chin plate on jaw relations. Due to the cushioning effect of the soft tissue^38^, we observed a discrepancy in the three records of jaw relations of each patient, even though the dentist instructed the patient to place their chin on the plates with the same level of pressure each time. The design of the next generation for chin plate may require a pressure-measuring instrument that can monitor the pressure of the soft tissue on the chin plate. If the pressure changes too much or too fast, it may indicate that the jaw relations changed during the regulating, headgear fixing, or scanning process.

Using the midline, lines of the angulus oris, and inferior margin of the upper lip, which were deduced from the lower one-third 3D facial data, obtained without dentures or occlusal bases in the mouth, was not sufficiently accurate for the final complete denture production. Using a virtual prediction software module^39,40^, it will be possible to predict the aesthetic reconstruction effects of wearing complete dentures in the lower one-third of the face of edentulous patients. The nasolabial angle (with proper lip support), the midline, lines of the angulus oris, and the inferior margin of the upper lip can be extracted from the predicted effects, which can facilitate the 3D design of the base and the arrangement of the teeth in dentures. Furthermore, with the help of 3D printing^41–44^ (which has the advantages of high efficiency and low cost), a complete denture can be produced and the distribution of the occlusal contacts of the artificial teeth analyzed using the T-scan.

This pilot study presents an approach for digital, direct recording of edentulous jaw relations using a handheld scanner and specially designed headgear without requiring occlusal bases and radiation. There have been no previous reports on digital and direct recording of jaw relation. Our findings show that it is possible to make a digital recording of edentulous jaw relation. There were marked individual differences in edentulous patients and limitations to the custom-developed headgear in our pilot study. Thus, better-designed scientific studies are required to improve the accuracy of digital recording of edentulous jaw relations.
